# Bluetongue Epidemiology in the European Union

**DOI:** 10.3201/eid1404.071441

**Published:** 2008-04

**Authors:** Claude Saegerman, Dirk Berkvens, Philip S. Mellor

**Affiliations:** * University of Liège, Liège, Belgium; † Institute of Tropical Medicine, Antwerp, Belgium; ‡ Institute for Animal Health, Pirbright, Surrey, UK

## Abstract

Bluetongue (BT) is a reportable disease of considerable socioeconomic concern and of major importance in the international trade of animals and animal products. Before 1998, BT was considered an exotic disease in Europe. From 1998 through 2005, at least 6 BT virus strains belonging to 5 serotypes (BTV-1, BTV-2, BTV-4, BTV-9, and BTV-16) were continuously present in the Mediterranean Basin. Since August 2006, BTV-8 has caused a severe epizootic of BT in northern Europe. The widespread recrudescence and extension of BTV-8 infections in northern Europe during 2007 suggest that requirements for BTV establishment may now be fulfilled in this area. In addition, the radial extension of BTV-8 across Europe increases the risk for an encounter between this serotype and others, particularly those that occur in the Mediterranean Basin, where vector activity continues for more of the year. This condition increases the risk for reassortment of individual BTV gene segments.

## Abstract 2

Central and northern Europe are now at risk from bluetongue virus.

## Abstract 3

Keywords: Bluetongue, BTV-8, epidemiology, cattle, sheep, Europe, disease perspectives, vectorial capacity, vector competence, vaccination, perspective

Bluetongue (BT) is an infectious but noncontagious viral disease caused by *Bluetongue virus* (BTV). The virus belongs to the family *Reoviridae*, genus *Orbivirus*; there are 24 serotypes (*1*). The viral genome consists of 10 double-stranded RNA segments that encode for 4 nonstructural proteins (NS1, NS2, NS3, and NS3A) and 7 structural (VP1-VP7) proteins (*2*,*3*). BTV serotypes 1, 2, 3, 4, 6, and 10 have a high pathogenic index and high epidemic potential (*4*). However, a high genetic diversity of BTV exists that is a consequence of both drift (i.e., point mutations) and shift (i.e., reassortment of individual BTV gene segments) so pathogenicity even within a serotype may be highly variable (*5*).

BT is a World Organization for Animal Health reportable disease and is of considerable socioeconomic concern and of major importance in the international trade of animals and animal products (*4*). Before 1998, BT was considered an exotic disease in Europe with just a few sporadic incursions (e.g., Spain and Portugal from 1956 through 1960) (*6*).

Our aim in this article is to provide a synthesis and some perspectives of BT epidemiology in the European Union (EU) since BTV’s introduction in 1998. To this effect, we provide a short overview of the epidemiologic situation in Europe, followed by a brief description of the susceptible species, a discussion of the vectorial capacity and competence of the *Culicoides* spp. vectors, and an outline of the modes of introduction and mechanisms of amplification.

## Epidemiologic Situation in Europe

### BTV in EU, 1998–2005

**Figure 1:**
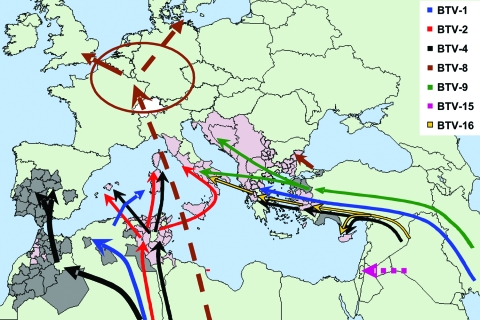
The molecular epidemiology of bluetongue virus (BTV) since 1998: routes of introduction of different serotypes and individual virus strains. *Presence of BTV-specific neutralizing antibodies in animals in Bulgaria, but the presence of BTV serotype 8 cannot yet be confirmed.

During this 8-year period, at least 6 BTV strains belonging to 5 serotypes (BTV-1, BTV-2, BTV-4, BTV-9, and BTV-16) have been continuously present in parts of the Mediterranean Basin, including several member states of the EU (Table, Figure 1) (*1*,*5*,*7*–*12*). This emergence of BT into parts of Europe never before affected was attributed mainly to climate change and was linked to the northern expansion of the major Old World vector *Culicoides imicola* (Kieffer), which is an Afro-Asiatic species of biting midge (*13*). Additionally, novel indigenous European vector species of *Culicoides* within the *Obsoletus* and *Pulicaris* complexes were involved.

**Table:** Outbreaks of bluetongue in Europe, 1998–2005*†

Country	Year of first outbreak	BTV serotype(s)	Main suspected or identified vector(s)
Albania	2002	9	*Culicoides obsoletus, C. pulicaris*
Bosnia–Herzegovina	2002	9	ND
Bulgaria	1999	9	*C. obsoletus, C. pulicaris*
Croatia	2001	9, 16	*C. obsoletus, C. scoticus*
Cyprus	2003	16	*C. imicola, C. obsoletus,*
Former Yugoslav Republic of Macedonia	2001	9	ND
France (Corsica)	2000	2, 4, 16‡	*C. imicola, C. pulicaris, C. obsoletus*
Greece	1998	1, 4, 9, 16	*C. imicola, C. obsoletus*
Italy	2000	1, 2, 4, 9, 16	*C. imicola, C. obsoletus, C. pulicaris*
Kosovo	2001	9	ND
Montenegro	2001	9	ND
Portugal	2004	2,§ 4	*C. imicola, C. obsoletus, C. pulicaris*
Serbia	2001	9	ND
Spain	2000	2	*C. imicola,C. obsoletus, C. pulicaris*
Turkey	1998	4, 9, 16	*C. imicola,C. obsoletus, C. pulicaris*

*BTV, Bluetongue virus; ND, no data recorded. †Sources (*5*,*7*–*10*). ‡This is an insufficiently attenuated vaccine strain (*11*). §This strain is indistinguishable from Onderstepoort BTV-2 live attenuated vaccine strain (*7*).

In the Mediterranean Basin 2 epidemiologic systems seem to predominate. The first one is located in the eastern part of the basin, where serotypes 1, 4, 9, and 16 were identified. In this system, the BTV strains originated in the Near, Middle, or Far East. The vectors included other species of *Culicoides* in addition to *C. imicola*. This finding was deduced from the fact that the disease penetrated into areas where *C. imicola* does not occur (the Balkans and beyond) (*9*). The involvement of novel vectors was subsequently confirmed when the causative virus was isolated from mixed pools of 2 species, *C. obsoletus* (Meigen) and *C. scoticus* (Downes and Kettle), collected in central Italy (*14*) and from *C. pulicaris* (Linnaeus) in Sicily (*15*). The second epidemiologic system comprises the western part of the Mediterranean Basin, where serotypes BTV-1, BTV-2, BTV-4, and BTV-16 were identified and the main vector is *C. imicola*. Although the appearance of BTV serotype 16 in this *C. imicola* system is the result of the westward spread of the virus across Europe (*16*), it is of particular interest because of strong indications that the field virus may represent a reversion to virulence of the attenuated vaccine (e.g., in Corsica and in Sardinia in 2004, strains of BTV-16 isolated from the field were identical to the live attenuated monovalent vaccine strain) (*1*,*11*) (Table, Figure 1).

### BT in Central and Northern Europe, mid-August 2006 to late December 2007

**Figure 2:**
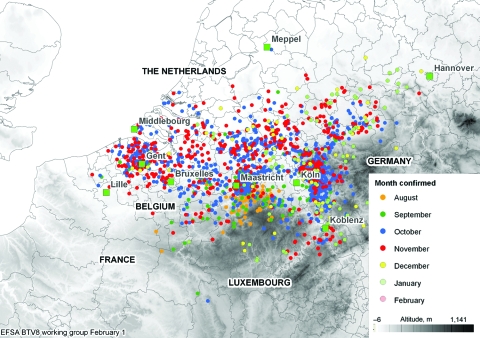
Monthly distribution of confirmed bluetongue virus 8 (BTV-8) outbreaks in northern and central Europe from August 17, 2006, through February 1, 2007. After January 1, 2007, few BTV cases were reported; those that were probably involved animals that had been infected, but not detected, in 2006.

**Figure 3:**
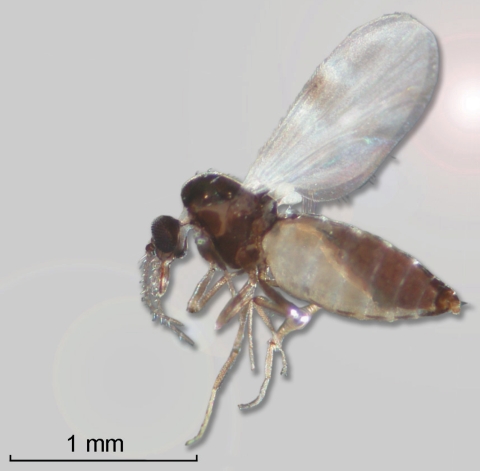
A gravid female *Culicoides dewulfi* collected from a location near bluetongue outbreaks in Belgium in 2006 (Photograph: Reginald De Deken and Maxime Madder, Institute of Tropical Medicine, Antwerp, Belgium).

BT was first identified in northern Europe in August 2006 and can be defined as an emergent disease in this zone (*17*). Between the date of the first report (August 17, 2006) and February 1, 2007 (*18*), 2,122 BT cases were entered into the European Commission’s Animal Disease Notification System (ADNS) (http://ec.europa.eu/food/animal/diseases/adns/index_en.htm) (Figure 2) (*19*). In this region, in 2006, a pool of 50 nonengorged, parous *C. dewulfi* (Goetghebuer) in the Netherlands were positive by PCR for BTV (*20*), and several pools of *C. obsoletus* complex in Germany (i.e., not identified down to species) were also PCR positive for BTV (*21*) (Figure 3). Although isolation of live BTV was not attempted in either instance, this research, conducted in an area where *C. imicola* does not occur, confirms the earlier findings of Mellor and Pitzolis, who isolated infectious BTV from nonengorged parous *C. obsoletus* in Cyprus, and shows that indigenous European *Culicoides* species can support a BT epizootic (*22*). Because *C. obsoletus* complex midges and *C. dewulfi* occur widely across central and northern Europe, this entire area must now be considered to be at risk for BTV (*23*,*24*).

Moreover, in relation to the demonstrated overwintering ability of the virus in northern Europe, small numbers of adult *Culicoides* spp. were captured in animal housing during the winter period (November 25, 2006, to March 9, 2007) (i.e., females of *C. obsoletus complex*, males of *C. obsoletus*, *C. scoticus*, and *C. dewulfi*) (*25*). Whether the occurrence of these midges and the possibility of their activity extending over the winter in such climatically protected locations can explain the persistence of virus from 1 vector transmission season to the next (*13*) or whether they represent newly emerged midges from nearby breeding sites is not known (*25*). Several hypotheses have been formulated to explain the overwintering ability of BTV: by persistence within surviving adult vectors themselves, transovarial transmission through the vector, or prolonged/persistent infection in viremic or aviremic vertebrate hosts (*13*,*25*,*26*).

**Figure 4:**
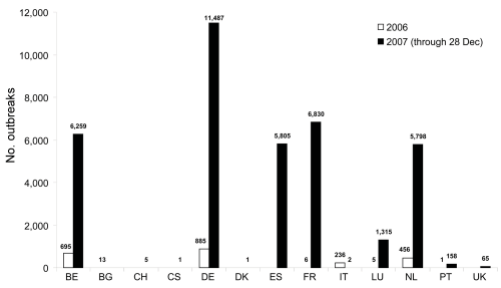
Number of bluetongue outbreaks in Europe since January 1, 2006 (all serotypes). BE, Belgium; BG, Bulgaria; CH, Switzerland; CS, Czech Republic; DE, Germany; DK, Denmark; ES, Spain; FR, France; IT, Italy; LU, Luxembourg; NL, Netherlands; PT, Portugal; UK, United Kingdom.

The focus of interest now is to see if BTV is able to survive regularly between vector seasons and become endemic to northern Europe. The recrudescence of BTV-8 in northern France, the Netherlands, Belgium, Luxembourg, and Germany in 2007, and also the emergence of BTV-8 in the United Kingdom, Denmark, Switzerland, and the Czech Republic, suggests that this may well be the case (*27*,*28*). Unlike farther south, where populations of the traditional vector, *C. imicola,* peak in the late summer and autumn, when most BT cases occur, populations of the indigenous European vectors peak earlier in the year; whether this will be reflected in a change in the temporal occurrence of BT cases remains to be seen. In the period from January 1, 2006, through December 28, 2007, 12 EU member states and Switzerland reported BT outbreaks on their territories, comprising all of the serotypes reported in Europe since 1998 (Figure 4) (*29*,*30*).

## Susceptible Species

BTV is transmitted between its ruminant hosts almost exclusively through the bites of the females of vector species of the *Culicoides* biting midge (*31*). The global distribution of BTV, therefore, is restricted to those regions where these vector species of *Culicoides* occur, and its transmission period is limited to the times when adult vectors are active. Depending on the species, adult vector activity generally starts some time in spring. Activity is positively correlated with temperature and reaches a maximum between 28°C and 30°C; activity decreases when the temperature drops and, for the traditional Afro-Asiatic vector *C. imicola*, is probably nonexistent at temperatures <10°C (*13*,*31*).

BTV can infect a broad spectrum of domestic and wild ruminants. However, serious clinical signs have been observed only in certain breeds of sheep (improved breeds) and a few deer species (*32*,*33*). Cattle and goats usually exhibit subclinical infections and therefore may serve as important and covert viral reservoirs for sheep (*32*). However, some serotypes such as serotype 8, which recently caused infection in northern Europe, exhibit a more important virulence in cattle (*34*,*35*) with serious socioeconomic consequences (*5*).

## Vector Capacity and Competence

Risk for BTV infection is linked closely to the presence of adult vector *Culicoides* spp (*31*). Until recently, *C. imicola* was believed to be the only important vector of BTV in southern Europe, but it is now known that several, newly recognized vector species are also involved. Others may be identified in the future.

Vector competence of an insect species and vector capacity of an insect population are important parameters in this respect (*36*). Vector competence is the (innate) ability of a vector to acquire a pathogen, maintain it, and successfully transmit it to a susceptible host (*13*). Vector competence may be determined in the laboratory by providing groups of insects of a particular species with blood meals of appropriate concentrations of virus and assessing infection and transmission rates. Vector competence is defined as the proportion of feeding insects that support virus replication and transmit virus after a suitable incubation period. In situations where transmission is difficult to demonstrate because of the technical problems in refeeding “difficult” insects such as *Culicoides* spp., it has become established practice to assume transmission if virus can be recovered from the salivary glands.

Vector capacity refers to the potential for virus transmission of an insect population and takes into account a range of insect, host, and environmental variables, including vector abundance, vector survival, biting and transmission rates, host preferences, and host abundances, under a range of external (e.g., bioclimatic) conditions. Vector capacity can be defined as the number of infective bites that an infected vector causes during its lifetime (usually 2–4 weeks in the case of vector species *Culicoides*) (*36*,*37*).

Determining the 2 parameters explained above is essential to accurately estimate vector transmission rates and predict whether BTV will become established in an area. Such detailed studies inevitably demand substantial financial and scientific resources and require a multidisciplinary approach.

## Modes of Introduction and Mechanisms of Amplification

Introduction of BTV from 1 area into another can occur in 4 ways: through animal movement (domestic and wild ruminants) or animal product transport (semen, embryos); by infected vector *Culicoides* spp. carried by various living (plants, animals) or inanimate (airplanes, ships) means; through the active flight of infected vector *Culicoides* spp. (local propagation); and through passive flight of infected vector *Culicoides* spp. on the wind (responsible for long-distance dissemination).

Whether the virus becomes established in a new area depends upon the number and distribution of susceptible hosts, the duration and titer of the BTV viremia in the hosts, the vector capacity of the local vector population, and the ambient temperature. In essence, establishment depends upon a sufficient number of vector *Culicoides* spp. becoming infected by feeding upon local viremic hosts, surviving long enough to ensure completion of the intrinsic incubation period (4–20 days, depending on ambient temperature), and transmitting the virus by bite to new hosts (*13*). The extrinsic incubation period is the interval between when a vector is infected and when it first becomes capable of transmitting the BTV to a new host (*38*). These requirements for BTV establishment have clearly been fulfilled in much of southern Europe, as BTV has survived there in many locations since the late 1990s.

## Conclusions

**Figure 5:**
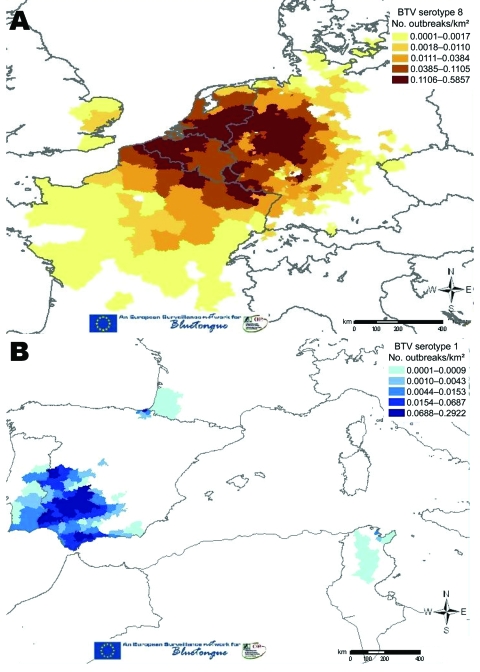
Number of bluetongue virus (BTV) outbreaks caused by BTV-8 (A) and BTV-1 (B) per kilometer (quartile scale) from May 1, 2007, to December 28, 2007 (EU-BTNET system; available from http://eubtnet.izs.it/btnet).

**Figure 6:**
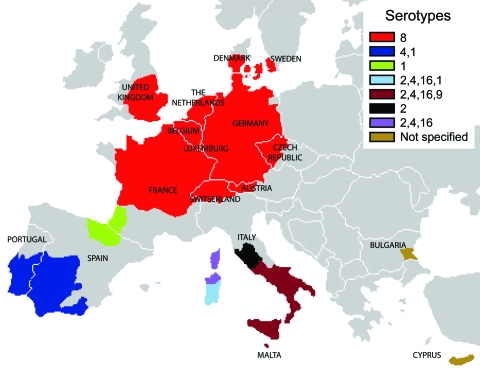
Bluetongue virus (BTV) restriction zones in Europe, by serotype. The radial extension of BTV-8 across Europe increases the risk for an encounter between this serotype and other serotypes that occur in the Mediterranean Basin (second epidemiologic system, where serotypes BTV-1, BTV-2, BTV-4, and BTV-16 were identified and the main vector is *Culicoides imicola*). This situation increases the risk for reassortment of individual BTV gene segments, and, in the more southerly areas, the period of vector activity is also likely to extend, leading to a longer BTV-8 season. In addition, BTV-1, which was first identified in sheep with clinical signs of BT in the south of the Iberian Peninsula in July 2007, has extended its range into northern Spain and southwestern France (Pyrénées-Atlantiques), since November 2007; this ongoing expansion is matter of major concern.

The widespread recrudescence of BTV-8 infections in northern France, Belgium, the Netherlands, Luxembourg, and Germany in 2007 and the emergence of BTV-8 in the United Kingdom, Denmark, Switzerland, and the Czech Republic in the same year suggest that the requirements for BTV establishment may now also be fulfilled in many more northerly and central parts of Europe (in the absence of *C. imicola*). In addition, the radial extension of BTV-8 across Europe (including the jump across the English Channel) (Figure 5) (*39*) increases the risk for an encounter between this serotype and others, particularly those that occur in the Mediterranean Basin (second epidemiologic system). BTV serotypes 1, 2, 4, and 16 have been identified in this area, and the addition of a further serotype will considerably increase the potential for reassortment between these viruses (Figure 6) (*27*,*40*). Indeed, the number of possible reassortments in the case of BTV, which has 10 segments, increases with the number of cocirculating serotypes (e.g., 1,024 for 2 serotypes [2^10^] and 59,049 for 3 serotypes [3^10^]) (*4*). Moreover, the phenomenon of reassortment has already been demonstrated during the 1998–2005 BTV outbreaks in Europe (*5*).

Furthermore, in the southern epidemiologic system, *C. imicola,* the Afro-Asiatic vector of BTV, occurs in addition to the *C. obsoletus* complex. As the population abundance of *C. imicola* peaks later in the year than the *Obsoletus* complex, this means that virus may be transmitted for a much greater portion of the year.

With regard to prophylaxis, possibly the best strategic option for control of clinical BT outbreaks in the European endemic areas is vaccination of susceptible animals with inactivated vaccines to protect against disease and to exclude the possibility of reversion to virulence of the vaccine viruses and reassortment between vaccine and field strains of the virus (*4*,*5*). Veterinary authorities and legislators throughout northern Europe would do well take note of these recent and considerable changes in the epidemiology of BT.
